# Genomic and Transcriptomic Insights into the Evolution and Divergence of MIKC-Type MADS-Box Genes in *Carica papaya*

**DOI:** 10.3390/ijms241814039

**Published:** 2023-09-13

**Authors:** Yunsu Dai, Yu Wang, Liwang Zeng, Ruizong Jia, Linwen He, Xueying Huang, Hui Zhao, Difa Liu, Haixu Zhao, Shuai Hu, Ling Gao, Anping Guo, Wei Xia, Changmian Ji

**Affiliations:** 1 Sanya Nanfan Research Institution of Hainan University, Sanya 572025, China; daiyunsu@163.com (Y.D.); wangyu7071@126.com (Y.W.); liudifa198707@126.com (D.L.); 2 Hainan Key Laboratory for Biosafety Monitoring and Molecular Breeding in Off-Season Reproduction Regions, Sanya Research Institute & Institute of Tropical Bioscience and Biotechnology, Chinese Academy of Tropical Agricultural Sciences, Haikou 571101, China; jiaruizong@itbb.org.cn (R.J.); Huangxymax@outlook.com (X.H.); zhaohui@itbb.org.cn (H.Z.); 17341989611@163.com (H.Z.); hushuai@itbb.org.cn (S.H.); gaoling_0898@163.com (L.G.); guoanping@itbb.org.cn (A.G.); 3 National Key Laboratory for Tropical Crop Breeding, Sanya 572025, China; 4 Key Laboratory of Applied Research on Tropical Crop Information Technology of Hainan Province, Institute of Scientific and Technical Information, Chinese Academy of Tropical Agricultural Sciences, Haikou 571101, China; 5 College of Marine Science, Hainan University, Haikou 570228, China; helinwen@hainanu.edu.cn; 6 Tropical Crops Genetic Resources Institute, Chinese Academy of Tropical Agricultural Sciences, Haikou 571101, China

**Keywords:** *Carica papaya*, type II MADS-box, evolutional scenario, duplicated genes, large-scale transcriptomes, expression divergence

## Abstract

MIKC-type MADS-box genes, also known as type II genes, play a crucial role in regulating the formation of floral organs and reproductive development in plants. However, the genome-wide identification and characterization of type II genes as well as a transcriptomic survey of their potential roles in *Carica papaya* remain unresolved. Here, we identified and characterized 24 type II genes in the *C. papaya* genome, and investigated their evolutional scenario and potential roles with a widespread expression profile. The type II genes were divided into thirteen subclades, and gene loss events likely occurred in papaya, as evidenced by the contracted member size of most subclades. Gene duplication mainly contributed to MIKC-type gene formation in papaya, and the duplicated gene pairs displayed prevalent expression divergence, implying the evolutionary significance of gene duplication in shaping the diversity of type II genes in papaya. A large-scale transcriptome analysis of 152 samples indicated that different subclasses of these genes showed distinct expression patterns in various tissues, biotic stress response, and abiotic stress response, reflecting their divergent functions. The hub-network of male and female flowers and qRT-PCR suggested that *TT16-3* and *AGL8* participated in male flower development and seed germination. Overall, this study provides valuable insights into the evolution and functions of MIKC-type genes in *C. papaya*.

## 1. Introduction

*Carica papaya* L. is globally recognized as one of the most prominent tropical crops. It exhibits distinct variations in fruit size and shape across various cultivated species [1]. This plant species has three distinct sex types, including female (XX), male (XY), and hermaphrodite ($XY^{h}$) [2]. Papaya is a diploid dicotyledonous plant with a relatively small genome size ranging from 350.3 to 351.5 Mb, and a gene content of 22,394 to 22,416 [3]. This plant species exhibits a plethora of remarkable traits, encompassing its perpetual year-round flowering capability, its facilitation of straightforward hybridizations, the proficient generation of considerable offspring, and reliable genetic transformation systems. These distinctive features collectively position it as an exemplary model species for studying tropical fruit and tree dynamics [4].

Based on the components of their protein domains, MADS domain proteins can be divided into two separate subgroups, type I and type II (MIKC-type), which are both important regulators in the entire process of gametophyte and embryonic development [5]. Type I predominantly consists of M domains, which are encoded by a DNA sequence approximately 180 bp in length [5,6,7]. Type II is mainly composed of M, I, K, and C domains, which together form the MIKC structure. Functionally, type I MADS-box genes predominantly govern the growth and development of female gametes and seeds, while type II MADS-box genes play vital roles in various developmental stages, including embryo development, flowering time regulation, and fruit development [8]. MADS-box genes participated in a diverse array of essential biological functions. In plants, MADS-box genes play a pivotal role in the regulation of various significant biological processes, encompassing flower, fruit, leaf, and root development [9]. MADS-box genes are instrumental in controlling flowering time, meristem characteristics, and the key components of the ABC organ characteristics model. At the heart of the ABC model lies a family of DNA-binding transcriptional regulators that are encoded by MADS-box genes, whereby the first round of A specifies the sepals, the second round of A+B specifies the petals, the third, B+C, round specifies the stamens, and the fourth round of C specifies the carpel alone. The A model has the *AP1* and *AP2* genes, the B model contains the *AP3* and *PI* genes, and the C model contains only one functional gene, *AG* [10].

Research investigating MIKC genes in *Cunninghamia lanceolata* (Chinese fir) revealed that most of the class B genes (*AP3/PI*) involved in the floral organ development were upregulated in male cones. Additionally, the *TM8* gene was upregulated in female cones, indicating that these genes may play a role in the sex development of Chinese fir [11]. The expression levels of type II genes in *Camellia chekiangoleosa* were much greater than those of type I genes [12]. An analysis of the transcriptomes under biotic stress in wheat (*Triticum aestivum*) revealed the differential expression of five MIKC genes in response to infection by *F. graminearum*, *S. tritici*, stripe rust, and powdery mildew. Specifically, the expression level of *TaMADS19* increased by 7 to 16 fold in wheat infected with *S. tritici*, while the expression of *TaMADS117* decreased by 3 to 7 fold in tissues infected with powdery mildew [13]. In the face of abiotic stress conditions, 10 MIKC genes exhibited altered expression patterns in response to forced phosphorus deficiency, drought, high temperature, and drought–heat cross-conditions. Specifically, under phosphorus deficiency, the expression levels of *TaMADS121*, *TaMADS93*, and *TaMADS21* were 4–7 times higher than the control group, while under heat stress, the expression levels of *TaMADS63* and *TaMADS41* were 1/50 and 1/12 of the control group, respectively [13].

Duplicated genes arise through evolutionary mechanisms such as genome-wide duplication, homologous recombination or transposon events, resulting in genes with extra copies in the genome. These duplicated genes may have similar functions, leading to the masking of phenotypic consequences of mutations in one gene by another, a phenomenon referred to as gene functional redundancy. The MADS-box family gene duplication provides abundant genetic material for the genetic diversity of transcription factors in flower organs, and can significantly accelerate evolution by providing new redundant genetic material that is unconstrained and free to evolve new functions [14]. Gene replication results in the synergistic expression of *APETALA3/PISTILLATA* with A and C function genes, respectively, to regulate B function in flower development, specifically petal and stamen functions [15]. The homologs of each duplicated gene pair retain comparable biochemical specificity but they differ in their gene expression patterns [16].

Despite the recognition of type II genes’ significance in papaya, an extensive exploration for their genome-wide distribution, characteristics, and evolution in the updated papaya genome, as well as their potential roles remain unresolved. The objective of this study is to fill the aforementioned research gap through the genome-wide identification and characterization of type II MADS-box genes, and to explore their evolutionary scenario and potential functions. A large transcriptome dataset with various tissues, developmental stages, as well as responses to biotic and abiotic stimuli was utilized to depict their expression profile and divergence, and unveil their potential functions. The findings could provide novel insights into the genomic landscape, evolution, and functions of type II MADS-box genes in *C. papaya*.

## 2. Results

### 2.1. Identification and Characterization of MADS-Box Type II Genes

**Figure 1:**
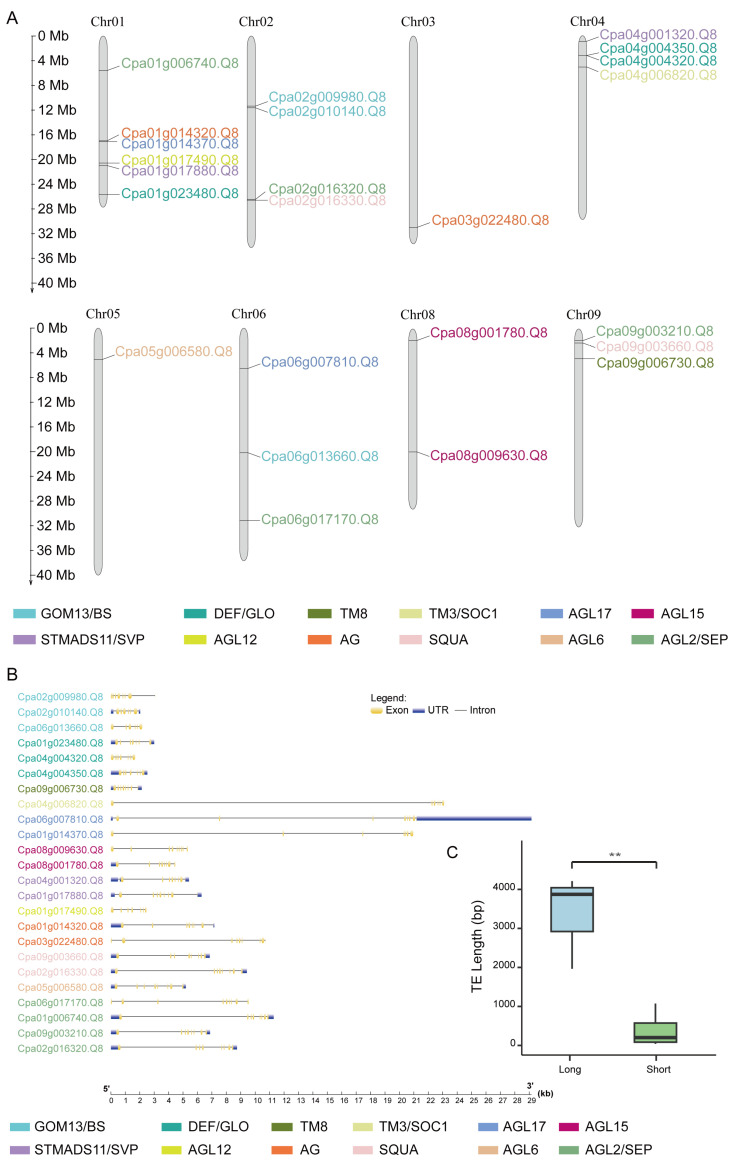
Thecharacteristics of twenty-four MADS-box Type II genes in *C. papaya*. (**A**) The chromosome location of twenty-four MADS-box Type II genes in the *C. papaya* genome. The differently colored gene IDs are represented by the twelve subfamilies of MADS-box Type II genes. Notably, there is no gene in subclade FLC. (**B**) Gene structures of twenty-four MADS-box Type II genes in *C. papaya*. Untranslated regions (UTRs) were colored purple, coding regions (CDS/exons) were colored yellow and non-coding regions (introns) were displayed as black lines. (**C**) The difference in TE content between long and short intron groups within the 24 MADS-box Type II genes. The Student’s *t*-test was used to determine whether the difference was significant. *p* value less than 0.01 is indicated by the symbol **.

**Figure 2:**
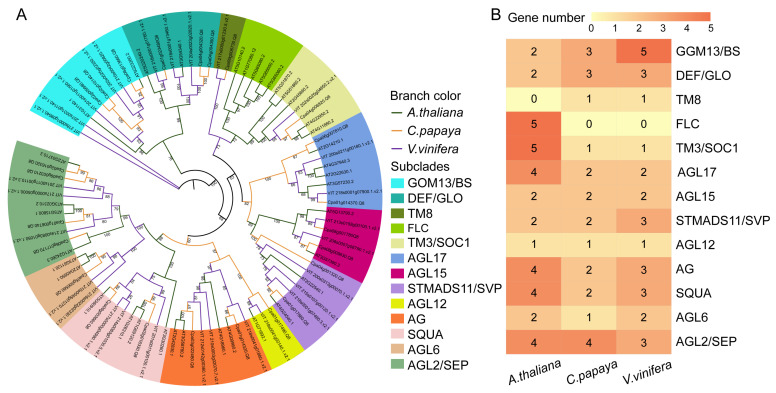
Evolution and classification of ninety MADS-box Type II genes. (**A**) Maximum-likelihood (ML) phylogenetic tree constructed using full-length protein sequences from *C. papaya*, *A. thaliana*, and *V. vinifera*. The MADS-box Type II genes of three species are represented by three different-colored branches (green: *A. thalina*; yellow: *C. papaya*; and purple: *V. vinifera*). GOM13/BS, DEF/GLO, TM8, FLC, TM3/SOC1, AGL17, AGL15, STMADS11/SVP, AGL12, AG, SQUA, AGL6, and AGL2/SEP are the 13 subclades of MADS-box Type II genes. The protein sequences of ninety Type II genes are provided in Table S1. The numbers in the phylogenetic tree nodes denoted the bootstrap values. (**B**) A heatmap depicted the number of genes in each MADS-box Type II subfamily.

In this study, we utilized the iTAK pipeline to examine a total of 90 MADS-box Type II genes from the genomes of *Vitis vinifera*, *Arabidopsis thaliana*, and *Carica papaya* (Table S1) [17]. Our analysis revealed that *C. papaya* had a lower number of identified genes (24) compared to *V. vinifera* (29) and *A. thaliana* (37). The gene names were named as their homologs in the *Arabidopsis thaliana* (Table S2). We further evaluated the physical and chemical properties of these 24 type II MADS-box genes using the online software Expasy [18] and WoLF PSORT [19] (Table 1, Figure 1). In *C. papaya*, the isoelectric point of the protein varied between 5.48 and 9.88. We found that four genes, namely *TT16-1*, *TT16-2*, *TT16-3*, and *AGL12*, exhibited an isoelectric point less than 8, while the remaining genes had an isoelectric point greater than 8 (Table 1, Figure S1). Different amino acids have different physical and chemical properties, and the activity and properties of proteins result from the interaction between their constituent amino acids [20]. We found that 24 genes in MADS-box Type II genes were clearly split into two subgroups according to their frequency distribution (Figure S1). Additionally, *TT16-1*, *TT16-2*, and *TT16-3* with isoelectric points less than 8 were found to be clustered into the same branch belonging to a GOM13/BS subclade in the phylogenetic tree, as well as *AGL12*, belonging to an AGL12 subclade (Figure 2A). The other genes with isoelectric points greater than 8 were clustered into other subclades. This observation implies that genes in the same subclade may prefer to keep similar isoelectric points. The DNA-binding domain (DBD) present in TFs is able to affect the diversity of their protein folds within and across organisms. Blanc-Mathieu et al. had reviewed and classified most of the 55 recognized plant TF types using the existing TFClass framework to organize all plant TFs’ types, following the TFClass hierarchy [21]. The crystal structure of the ORE1-NAC domain alone (Apo) and its DNA-binding form in *Arabidopsis thaliana* reveals that the DBD (ORE1-NAC) is crucial for DNA recognition [22]. The protein length of these genes ranged from 186 to 260 AAs, and their molecular weight ranged from 21,586.97 to 30,265.32 Da. Furthermore, a subcellular localization analysis indicated that all 24 genes were located in the nucleus, which is consistent with their transcript factor role.

The intron-exon organization of papaya’s MADS-box genes was analyzed using GSDSv2.0 [23] to explore their structural diversity. Exon gain or loss may contribute to changes in protein domain structure and protein function [24]. We investigated the MADS-box Type II genes’ structural characteristics and discovered that the exon numbers varied widely in papaya, which was primarily distributed between six and nine (Figure 1B). An increased number of exons could result in more isoforms, as is the case for the Cpa08g009630.Q8 and Cpa03g022480.Q8, which have a large number of exons (nine exons) (Figure 1B, Table S3). We also observed the extremely long gene lengths of Cpa04g006820.Q8, Cpa06g007810.Q8, and Cpa01g014370.Q8 with normal exon numbers, which were caused by their abnormally large introns (Figure 1B). Retrotransposons have played an important role in the evolution of plant genomes [25,26]. Ginkgo displayed the longest average intron length, with introns in Ginkgo characterized by many repeat-element insertions [27]. However, the sources of ultra-large introns in MADS-box Type II genes of papaya remain unclear. To investigate this phenomenon, we conducted transposon element annotation for these large introns. Remarkably, our analysis revealed that long terminal repeat (LTR) elements predominantly contributed to the expansion of these introns (Figure 1C, Table S4).

### 2.2. Phylogenetic Relationship and Conserved Motif Organization of MADS-Box Type II Genes

To examine the phylogenetic relationships of these genes, we constructed the maximum-likelihood (ML) tree using IQ-TREE v2.1.2 [28] (Figure 2A).

The resulting phylogenetic tree showed that the MADS-box Type II genes in papaya were clearly divided into thirteen subclades, which were assigned to the orthologous nomenclature of their counterparts in *A. thaliana*, including GOM13/BS, DEF/GLO, TM8, FLC, TM3/SOC1, AGL17, AGL15, STMADS11/SVP, AGL12, AG, SQUA, AGL6, and AGL2/SEP. This result was consistent with that of previous studies [8,29]. Remarkably, in *C. papaya*, a number of subclades showed a reduced size, indicating the presence of gene loss events in the papaya genome (Figure 2B). TM3/SOC1 subclade contained only one copy in *V. vinifera* and *C. papaya*, while five copies in *A. thaliana* (Figure 2A). The TM3/SOC1 homolog in Medicago promotes flowering and primary stem elongation [30], while in tomato, it positively regulated the inflorescence architecture [31]. We focused on AGL12, which plays an important role in root development [32] and alkaloid biosynthesis [33]. This subclade contained only one copy in all of *A. thaliana*, *V. vinifera*, and *C. papaya*, implying its housekeeping role in eudicots. Notably, we found that the AGL2 subclade was expanded in *C. papaya*, with five members distributed on different chromosomes (Figure 1A). This subclade is known to play a fundamental role in the development of all floral organs, seeds, embryos [34], and stamens [35]. Its expansion in *C. papaya* may contribute to the floral diversity and complexity of sexuality determination [36].

Furthermore, we used the MEME tool in MEME suit [37] (https://meme-suite.or-g/meme/tools/meme, accessed on 9 July 2023) to analyze the conserved motif organization of MADS-box Type II genes in different plant species (Figure S2A and Table S5). A total of 10 conserved motifs were identified from 90 Type II protein sequences (Figure S2B). Consistent with the phylogenetic tree, members belonging to the same subclade displayed a nearly similar composition of motifs (Figure 2A and Figure S2A,B). Motif 1 (MADS domain) was present in nearly all protein sequences, except for *TT16-1*, which belongs to the GOM13/BS subclade (Figure S2A,B). These results provide valuable insights into the evolutionary relationships and conserved motif organization of MADS-box Type II genes in papaya and other plant species. The complex structures of these genes may be helpful for their involvement in various growth and development processes in plants [38].

### 2.3. Genomic Distribution of MADS-Box Type II Gene

Based on their physical locations, we mapped the MADS-box Type II genes onto the respective chromosomes of *C. papaya* in order to illustrate the genomic distribution of these genes in papaya (Figure 1A). Except for Chr07, we discovered that Type II genes were extensively dispersed on practically all chromosomes. Chr01 had the greatest amount of Type II genes, which belonged to six separate subclades. The median gene numbers on chromosomes of Chr02, Chr04, Chr06, Chr08, and Chr09 ranged from two to four, while Chr03 and Chr05 had relatively fewer genes (Figure 1A). Notably, the Type II genes tended to be located disproportionally on the distal regions of the chromosomes (Figure 1A). Interestingly, we observed many adjacent gene pairs, which may have been generated by duplicated events (Figure 1A). An example of such a gene pair would be the near chromosomal locations of *AP3-1* and *AP3-2* on Chr04, which belong to the same subclade (Figure 1A).

### 2.4. Evolutionary Scenario of the MADS-Box Type II Subfamily

Gene duplication is frequently associated with gene family expansion, which is seen as a crucial driver in gene family evolution [39,40], leading to genetic diversity [41]. In *A. thaliana*, almost 90% of regulatory genes were found to have increased in number by three whole-genome duplication (WGD) events [42]. In this study, we identified 15 duplicated pairs of MADS-box Type II genes in papaya. Transposon duplicated genes accounted for 66.7% (10) of the total, with one proximal duplicated gene, two whole-genome duplicated genes and two dispersed duplicated genes (Table 2).

The result suggested that gene duplication is a major force that shapes the MADS-box Type II subfamily in papaya, with approximately 83.3% of MADS-box Type II genes resulting from gene duplication. The occurrence time of these duplicated genes ranged from 26.1 Mya to 1.4 Mya, with *AGL18* and *AGL21-1* being the earliest duplicated genes (25.9 Mya) and *AP3-1* and *AP3-2* the youngest duplicated genes (1.4 Mya) (Table 2). The non-synonymous/synonymous substitution ratio values (Ka/Ks) of these gene pairs indicated that these gene pairs experienced strong purifying during papaya evolution (Table 2). Previous research showed that 95.4% (103) of the 118 MADS-box Type II genes in blueberry were generated from duplication events [43,44]. Most of these duplicated genes have Ka/Ks values less than 1, implying purifying selection pressure on them [43]. We found that all Type II duplicated gene pairs in papaya displayed low Ka/Ks values, all being less than 1, indicating that purifying selection also occurred on these duplication-derived gene pairs. Papaya only experienced a whole-genome triploidization event (γ-WGT) in the common ancestor eudicot karyotype (AEK) at 120–125 mya without any additional duplication events [45,46,47]. We found that the duplications of *AP1/AGL8* and *AGL24/SVP* resulted from γ-WGT.

**Figure 3:**
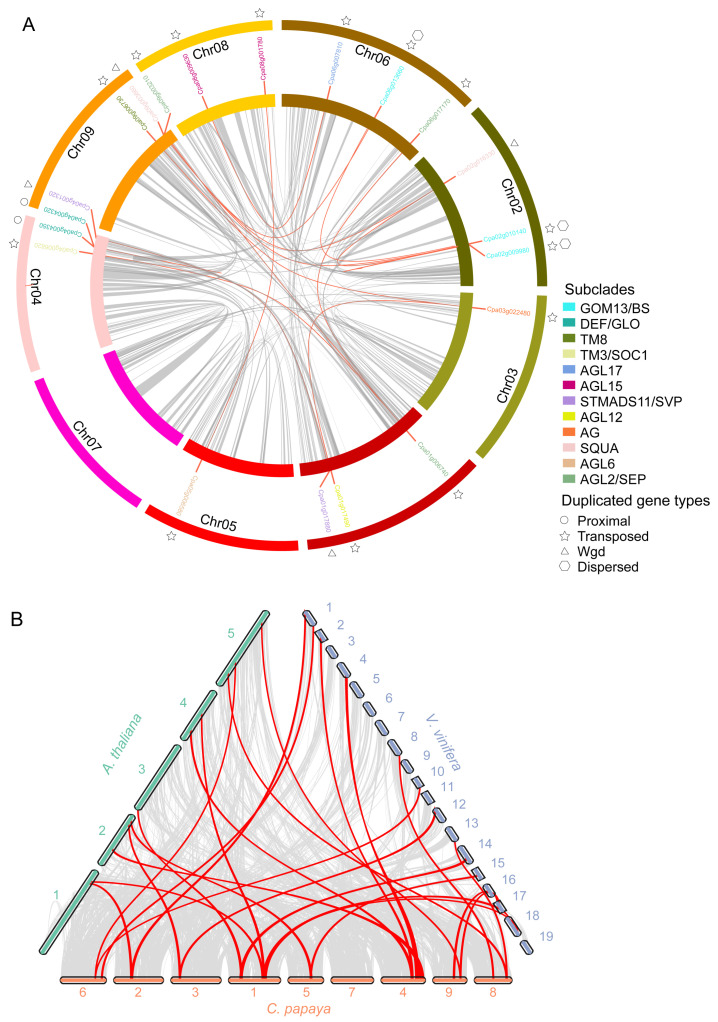
Duplicated gene pairs of the MADS-box Type II subfamily on papaya genome. (**A**) The genomic landscape of duplicated gene pairs of Type II genes in the papaya genome. The nine papaya chromosomes are represented by colored tracks. The gray lines represent the papaya’s internal collinear blocks. The red lines indicate the gene pairs that have been duplicated in the Type II subfamily. Outside the chromosomal tracks, three different symbols represent three different duplication forms of proximal, transposed, and wgd origin. (**B**) Synteny plot of MADS-box Type II genes among *C. papaya*, *A. thaliana*, and *V. Vinifera*. The collinear blocks of *C. papaya*, *A. thaliana*, and *V. Vinifera* are represented by gray lines, whereas the syntenic relationships of the MADS-box Type II gene pairs are represented by red lines.

To visualize the loci relationship of MADS-box Type II duplicated genes, we connected each duplicated gene pair with a red line in the papaya genome (Figure 3A).

Our analysis revealed that 84% of duplicated genes (20) were mapped onto eight chromosomes, except for Chr07. We found that most duplicated gene pairs presented on different chromosomes (Figure 3A). We also identified 14 orthologous gene pairs between *C. papaya* and *V. vinifera* (red lines), as well as 11 orthologous gene pairs between *C. papaya* and *A. thaliana* (Figure 3B).

### 2.5. Expression Analysis of Papaya Type II Genes in Different Tissues

**Figure 4:**
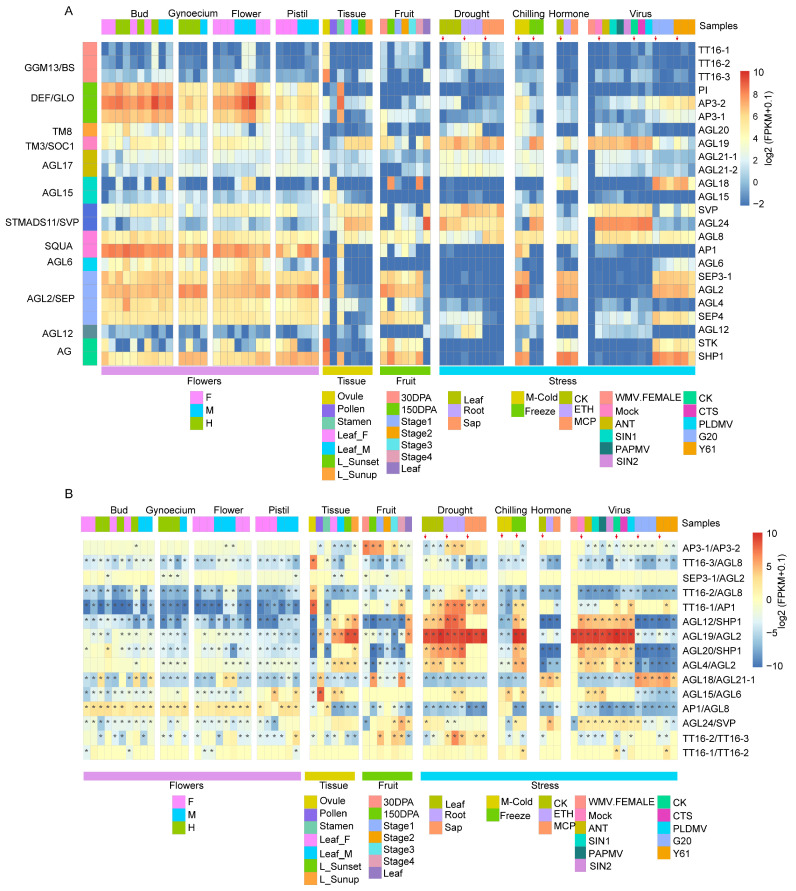
Expression profiles and divergence of MADS-box Type II genes. (**A**) Transcriptomic survey of MADS-box Type II genes among 73 transcriptome samples. These datasets included transcriptomes in different tissues (flower, stamen, pistil, pollen, and leaf) under abiotic stress (drought stress, chilling stress, and hormone stress) and under biotic stress (*Papaya Mosaic virus*: PapMV; *Papaya Ringspot virus*: PRSV; *Papaya leaf-distortion mosaic virus*: PLDMV; and pathogen (Anthracnose: *Colletotrichum brevisporum*). The colored bands above the heatmap represented the sample information, whereas the colored bands at the bottom of the heatmap indicate the group information. The red arrows above the heatmap represent the beginning (0 h or 0 day) of stress treatments. (**B**) Expression divergence of duplicated gene pairs in different transcriptome datasets. The asterisks indicate that the expression changes between the duplicated gene pairs are larger than a two-fold change.

To explore the potential role of Type II genes in different tissues, we investigated the expression levels of 24 MADS-box Type II genes in various tissues, including male flower, female flower, hermaphrodite flower, pistil, ovule, pollen, stamen, and leaf (Figure 4A, Tables S6 and S7).

Our analysis revealed that members of the GGM13 subclade exhibited low expression levels in most tissues, but were highly expressed in the male flower and ovule, indicating their crucial biological role in male flower development and ovule formation (Figure 4A). Conversely, members of the DEF/GLO, SQUA, and AGL2 subclades displayed high expression levels in most examined tissues, except for pollen, stamen, and leaf. *AGL19*—which belonged to the TM3/SOC1, SVP, and *AGL24*—which in turn belonged to the HTMADS11—as well as *AGL4*—which belonged to the AGL2 subclade—were highly expressed in the leaf (Figure 4A; Table S8). We observed that *AP3-1*, *AP3-2*, *AGL2*, *AGL24*, *STK*, and *AGL18* were the most highly expressed genes in flower bud, pistil, leaf, ovule, pollen, and stamen, respectively, implying their important role in tissue differentiation (Figure 4A). Additionally, we analyzed the expression profiles of type II MADS-box genes in papaya male, female, and hermaphrodite flowers and found that most of them are highly expressed in different sexual tissues without obvious differences in expression levels among three sexual flowers (Figure S3). These results indicated that type II MADS-box genes played a key role in the different floral organ developments of *C. papaya*, which is consistent with previous studies [11]. Intriguingly, we found that *AGL18*, which originated from the gene duplication of *AGL21-1*, exhibited high expressions in male/hermaphrodite buds, pollens, and male flowers but is lowly expressed in female buds and flowers, indicating an important role of *AGL18* in papaya male flower development. In contrast, *AGL21-1* showed an opposite expression pattern (Figure 4A). The observed expression divergence between *AGL18* and *AGL21-1* in different sexual organs indicated functional differentiation between them during the development of male and female flowers. This finding provides valuable insight into the transcriptomic changes of type II MADS-box genes, underlying the development of papaya’s sexual organs.

### 2.6. Expression Analysis of Papaya Type II Genes under Abiotic and Biotic Stresses

We characterized the expression profile of type II genes under abiotic stresses including drought and chilling (Figure 4A, Tables S6 and S7). Our analysis revealed that *AGL19*, belonging to the TM3/SOC1 subclade, exhibited high expression levels under drought and chilling stresses, similar to the expression pattern observed for genes in the STMADS11 subclade (Figure 4A, Tables S6 and S7). *AGL19* is a transcription factor that may promote flowering, especially in conditions where a short cold vernalization response is required [48]. In a study on rice, the expression levels of *OsMADS18* (SQUA), *OsMADS22* (STMADS11), *OsMADS26* (AGL12), and *OsMADS27* (AGL17) genes were upregulated by more than two-fold in response to cold and dehydration stresses [49]. Therefore, members of these four subclades may play an important role in cold and dehydration stress response. This result was also verified in the expression profile of papaya. Members of the STMADS11/SVP subclade were significantly highly expressed under chilling and dehydration stresses (Figure 4A, Tables S6 and S7), suggesting their involvement in cold and drought stress resistance in papaya. In SQUA subclade, *AGL8* was found to be expressed in dry saps. *AGL12* and *AGL17* were expressed at low levels in drought-stressed leaves, which is inconsistent with the results observed in rice [49].

To investigate the role of type II genes under biotic stress, we analyzed the expression profiles of these genes in leaves that had been infected with virus (*Papaya Mosaic virus*: PapMV, *Papaya Ringspot virus*: PRSV, and *Papaya leaf-distortion mosaic virus*: PLDMV) and pathogen (Anthracnose: *Colletotrichum brevisporum*) (Figure 4A, Tables S6 and S7). Our findings revealed that the expression level of the *AGL19* gene in the TM3/SOC1 subclade was significantly downregulated, following infection with the *Papaya leaf-distortion mosaic virus* (Figure 4A, Tables S6 and S7). Furthermore, following 0 h, 24 h, and 48 h of *Colletotrichum brevisporum* inoculation, *SVP* gene in the STMADS11/SVP subclade considerably increased expression levels. In contrast to the control group (0 h after inoculation), the expression level of the *SEP4* gene in the AGL2/SEP subclade was downregulated in leaves with *C. brevisporum* infection (Figure 4A, Tables S6 and S7). These results implied that the type II genes in papaya may be involved in biotic stress response, and examples of *AGL19* and *SEP4* members of the AG subclade may play a role in papaya’s resistance to Anthracnose stress.

### 2.7. Expression Divergence of Papaya Type II Duplicated Genes

The above analysis showed that gene duplication majorly contributed to the MADS-box Type II family in papaya. We investigated the potential functional divergence of duplicated genes by examining the expression changes of type II duplicated genes in a large transcriptome dataset, which included 152 samples from various tissues, abiotic, and biotic stresses (Tables S6 and S7). Our findings demonstrated that the majority of duplicated gene pairs displayed an apparent expression bias in different tissues, under various abiotic stresses and different pathogen infections, implying functional differentiation during their evolution process after duplication occurrence (Figure 4B and Table S9). Notably, *AGL19* and *AGL2* displayed a conserved expression divergence in most of the surveyed transcriptomes, especially under drought stress, chilling stress, and different virus infections. The high sequence divergence (47.7% sequence identity between them) has resulted in functional divergence of *AGL19* and *AGL2* (Figure 4B). *AP1* and *AGL8* duplicated gene pairs revealed an altered direction of expression bias between flower-related organs and stress-treated samples (drought, chilling, hormone, virus, and fungus) in contrast to other duplicated genes with a conserved direction of expression bias in most samples (Figure 4B). The expression levels of the *AP1* gene was higher than those of *AGL8* in different tissues (flower, stamen, and pollen), and under hormone treatments (ETH and MCP). However, under abiotic stresses (drought and chilling) and different pathogen infections (*Papaya Mosaic virus*, *Papaya Ringspot virus*, and Antagonism), the expression levels of *AP1* were lower compared to *AGL8* (Figure 4B). Additionally, *TT16-3* was highly expressed in embryo but lowly expressed in other tissues. *AGL8*, which originated from gene duplication from *TT16-3*, showed low expression levels in ovule and pollen, but high expression in other tissues (Figure 4B and Figure S3). Overall, we concluded that the expression bias of type II duplicated genes was widespread and conserved in papaya, indicating the functional differentiation of the duplicated genes.

### 2.8. Type II Genes Associated with Papaya Male Flower Development

**Figure 5:**
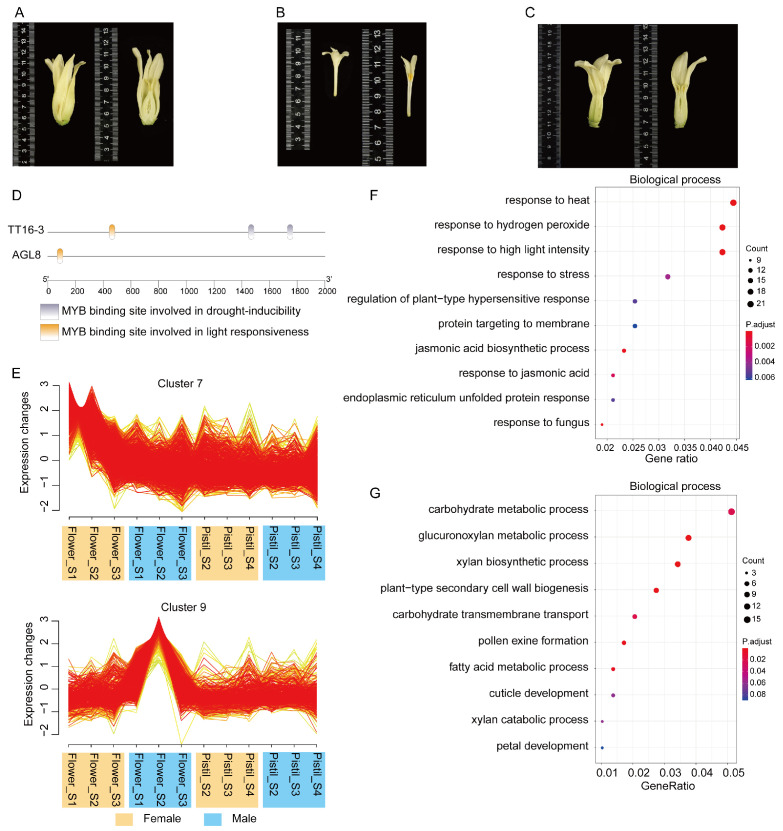
Gene expression dynamics among different stages of flower development. Phenotypes of papaya (**A**) female, (**B**) male, and (**C**) hermaphrodite flowers. (**D**) Cis-regulatory element analysis of *AGL8* and *TT16-3*. The MYB domain was one of the cis-acting elements found in the regulatory regions of *AGL8* and *TT16-3*. (**E**) Mfuzz clustering analysis of differentially expressed genes alongside the different development stages of flower and pistil. Cluster-7 and Cluster-9, which contained *AGL8* and *TT16-3*, are shown in (**F**) GO enrichment analysis of Cluster-7 genes. (**G**) GO enrichment analysis of Cluster-9 genes.

Papaya is a trioecious plant species with three sex types: female, male, and hermaphrodite flowers (Figure 5A–C).

In plants, MADS-box genes-regulated flower development [9] and type II MADS-box genes have been associated with flowering time regulation [8]. However, the potential role of Type II genes in different sexual flowers is still unclear. Here, we analyzed the transcriptome data of papaya male and female flowers at three different development stages. A total of 7159 differentially expressed genes (DEGs) were identified between male and female flowers at three development stages, including 15 type II genes (Table S10a). We found that these DEGs were significantly enriched in biological processes of pollen tube growth, jasmonic acid-mediated signaling pathway, and response to karrikin (Figure S4A,B). To obtain the gene set associated with type II genes in male and female flower development processes, we present a time-series analysis of the transcriptomes from flowers of different sex types and pistils at three development stages using Muffz package (Figure 5E and Figure S5). We found that Type II gene *AGL8* was located in Cluster-7, which exhibited a sharply increased expression pattern in male flowers, while Type II gene *TT16-3* was contained in Cluster-9, which displayed a gradual decreasing expression trend in female flowers (Figure 5E). Cis-acting elements analysis for 2000 bp upstream of CDS sequences revealed that MeJA responsiveness, light responsiveness, auxin responsiveness, drought responsiveness, low temperature responsiveness, and the MYB binding site were abundant on the promoter regions of *AGL8* and *TT16-3*, implying their potential role in the stress response and plant hormone signaling pathway (Figure 5D and Figure S6). The analysis of cis-acting elements in the promoters of 24 type II genes showed that these elements were mainly involved in low-temperature seed-specific regulation, abscisic acid, auxin, and endosperm expression, and gibberellin response (Figure S6). We performed the motifs annotation for all promoters of 22,657 protein-coding genes, including 24 MADS-box Type II genes using FIMO. Then, the enrichment analysis of TFBS motifs for 24 MADS-box Type II genes were examined by comparing all the TFBS motifs generated from all papaya protein-coding genes. We used the Fisher exact test to estimate the significance of TFBS motifs’ enrichment in MADS-box Type II genes. The TFBS motifs that exhibited adjusted *p*-values below 0.05 were regarded as putative regulatory transcription factors (TFs) for the Type II genes in papaya, indicating their potential involvement in gene regulation. Finally, we obtained 19 potential regulators for 24 MADS-box Type II genes in papaya (Table S11). We found that five TFs and their corresponding eleven potential target MADS-box Type II genes commonly displayed different expressions between male and female flowers, implying that the transcription regulatory relationships between them involved different sex-type flower developments (Table S10b). The TF genes are *BZIP42*, *BZIP44*, *BZIP53*, *CDF5*, *DOF3.6*, *CDF5*, and *TCX2*. Transcriptomic analysis showed that the enriched *BZIP42*, *BZIP44*, *BZIP53*, and the *AP3-2* of the MADS-box Type II gene exhibited different expression levels between male and female flowers at the second development stage. Notably, *BZIP42* and *AP3-2* are differently expressed between male and female flowers across three development stages, implying the important role in different sex-type flower differentiation in papaya. *DoF3.6* is differently expressed with nine MADS-box Type II genes, including *AGL24*, *PI*, *TT16-1*, and *TT16-2* (Table S10b). The observation suggested the complex regulatory relationships in papaya MADS-box Type II genes. We identified twelve MADS-box transcription factors (TFs) in this list: *AGL15*, *SOC1*, *AP1*, *PI*, *AGL6*, *AGL13*, *FLC*, *AGL16*, *AGL27*, *AP3*, *SVP*, and *FLC*. According to the BioGRID database (https://thebiogrid.org/, accessed on 11 August 2023), the vital transcription factors *PI*, *AGL6*, and *AGL16* have been identified as being associated with the MADS-box type II genes. Through a FIMO prediction of the 24 MADS-box Type II genes, it was observed that *PI* interacts with *AP3* [50], and a subsequent transcriptome analysis further demonstrated the positive regulatory role of *PI* on *AP3*. *AGL6* exhibits interactions with *AGL20* and *AGL24* [51]. During the second stage of male and female development, *AGL6* exerts a negative regulation on *AGL20* while positively influencing *AGL24*. Subsequently, in the third stage of male and female development, *AGL6* exhibits negative regulation on both *AGL20* and *AGL24*. *AGL16* displays interactions with *AGL6*, *AGL20*, and *AGL24* [52], and during the third stage of male and female development, it exerts a positive regulation on *AGL20* and *AGL24* while exerting a negative regulation on *AGL6*. GO term and KEGG pathway enrichment analyses of genes in Cluster-7 (Figure 5F and Figure S4D) and Cluster-9 (Figure 5G and Figure S4E) also supported the potential co-expressed genes associated with hormone metabolism and stress response function in Cluster-7 and Cluster-9.

**Figure 6:**
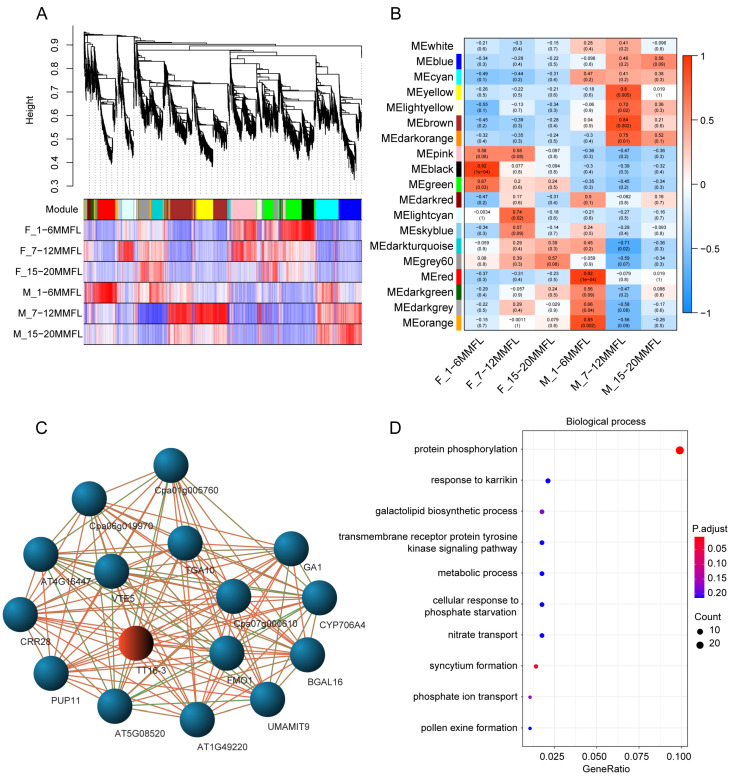
WGCNA of the differently expressed genes in papaya male and female flowers at different development stages. (**A**) Dendrogram of WGCNA-identified co-expression modules in six transcriptome datasets from different flower development stages. Each leaf on the tree represents a single gene. Nineteen co-expression modules were built, each identified with a different color. (**B**) Correlation between modules and traits. Flower development stages were set as trait values to identify key modules involved in corresponding development stages. Each row represents one module, and each column indicates a distinct trait. The correlation coefficient between the module and the trait is represented by the value in each cell at the row–column intersection, which is depicted using the color scale on the right. The value in parentheses in each cell represents the p value. The correlation coefficient between the module and the trait is indicated by the color of each grid at the row–column intersection. (**C**) Core hub-network of co-expressed genes associated with *TT16-3* in the cyan module. The weight values of the linked lines from low to high are highlighted by the colors ranging from green to orange. (**D**) GO enrichment analysis of genes in the cyan module.

To further explore the hub-genes and hub-networks related to the duplicated genes of *AGL8* and *TT16-3*, we fulfilled a weighted gene co-expression network analysis (WGCNA) for differently expressed genes between male and female flowers at three development stages (Figure 6A).

Modules and traits correlation analysis showed that many modules are specifically involved in male or female flower development (Figure 6B). We focused on the cyan modules, which included gene *TT16-3* and significantly correlated with male flower development (Figure 6B). *AGL8* is in the green module. According to the enrichment analysis, genes in the cyan module were mostly enriched in plant hormone signal transduction and phenylpropanoid biosynthesis (Figure 6D and Figure S4C). We chose 150 genes directly connected with *TT16-3* in the cyan module to construct the co-expressed networks of the hub-genes (Figure S7A). Many genes in the cyan module were discovered to be strongly expressed in the late development stage of female flowers, whereas the other genes were found to be substantially expressed in the early and medium development stages of male flowers (Figure S7A,B). Furthermore, we identified 15 key genes that co-expressed with *TT16-3* in the cyan WGCNA module (Figure 6C, Tables S12 and S13). The expression level of these hub-genes in male flowers was apparently higher than those in female flowers (Figure S7D), whereas nearly equal among other tissues (Figure S7C,E–G), indicating their role in differences between male and female flower development in papaya.

### 2.9. Relative Expression of *AGL8* and *TT16-3* in Seed Germination and Male Flower Development

**Figure 7:**
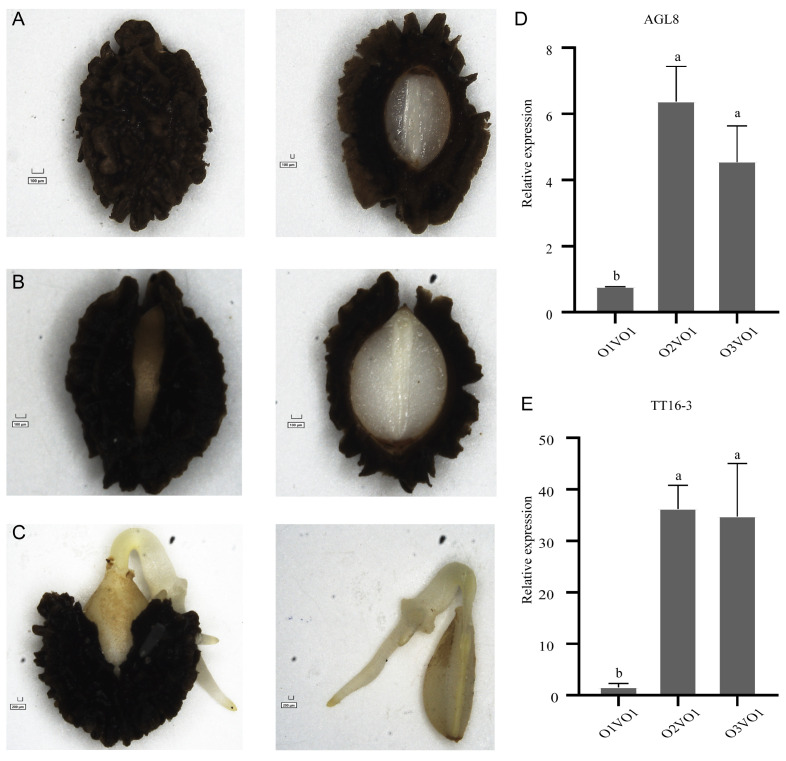
Papaya seed phenotypes at different stages of germination. Photos on the left are seed overviews of seed phenotypes, and on the right are sectional diagrams of seeds. (**A**) Germination of papaya seed at the first stage. (**B**) Germination of papaya seed at the second stage. (**C**) Germination of papaya seed at the third stage. (**D**,**E**) Relative expression levels of two duplicated genes (*AGL8*, and *TT16-3*) during three germination stages of papaya seeds. RNA was extracted from the seeds at 0, 96, and 192 h after germination. Expression levels of *AGL8* and *TT16-3* in papaya seeds at three germination stages were determined via qRT-PCR. The relative expression levels were calculated against the mean expression value of the internal reference gene papain. Error bars represent the standard deviations, and data are mean ± standard deviation. Three biological duplicates were designed for each experiment. Duncan’s multiple range tests were performed, and the significant differences at a significance level of *p* < 0.05 were indicated by different letters assigned to each value.

To examine the expression patterns of *AGL8* and *TT16-3* in seed germination, qRT-PCR was performed. Whole seeds of papaya at three different germination stages, namely early, middle, and late germination processes, were photographed using a microscope (Figure 7A–C).

**Figure 8:**
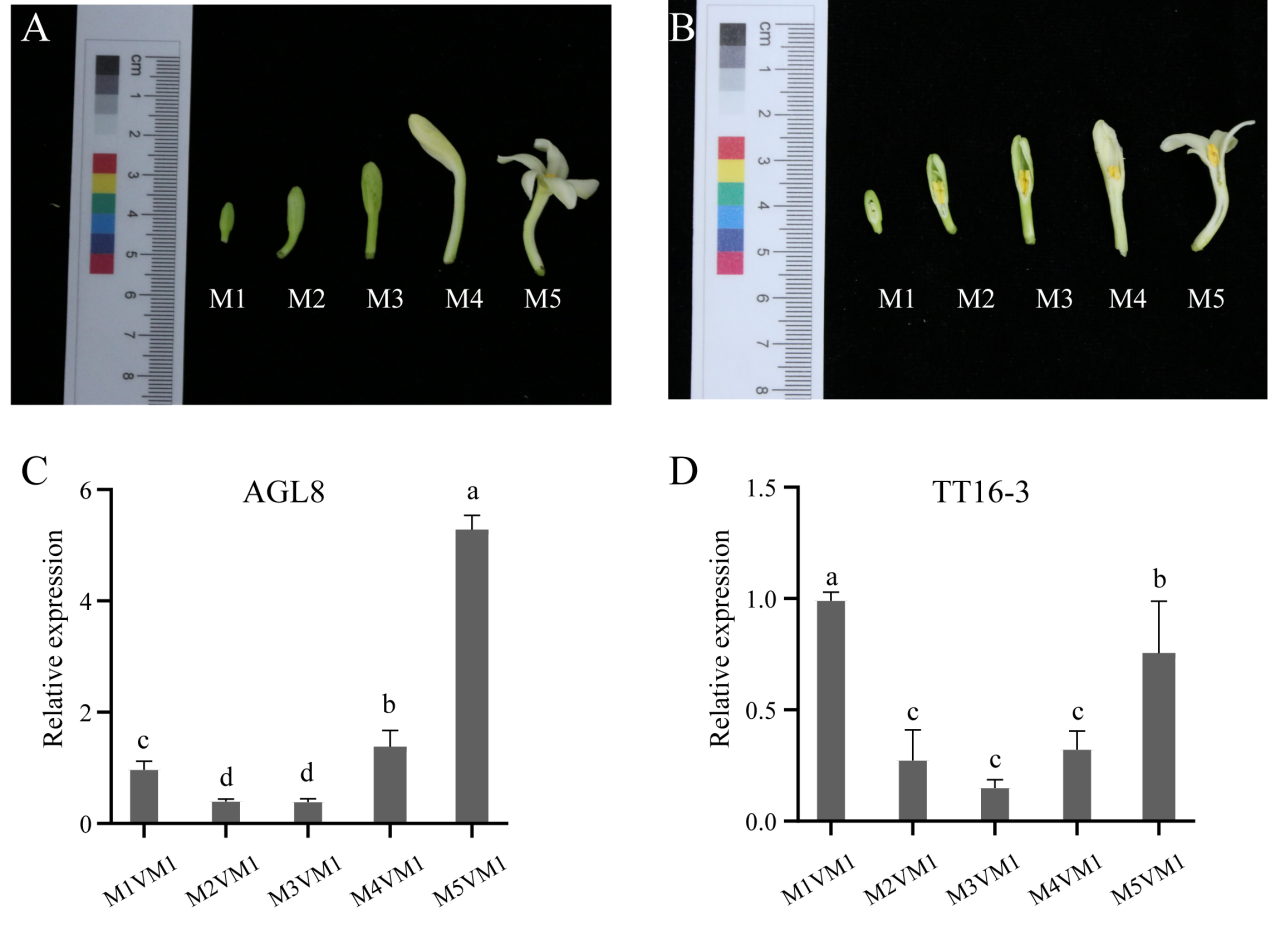
Overall and sectional diagrams of papaya male flowers at five development stages, and relative expression levels of two duplicated genes (*AGL8*, and *TT16-3*). (**A**) Overall photos of papaya male flowers at five development stages. (**B**) Sectional phenotypes of papaya male flowers at five development stages. (**C**,**D**) Relative expression levels of *AGL8* and *TT16-3* in papaya male flowers at five developmental stages. Expression levels of *AGL8* and *TT16-3* MIKC were determined via qRT-PCR. The relative expression levels are shown against the mean expression value of internal reference gene papain. Error bars represent the standard deviations, and data are mean ± standard deviation. Three biological duplicates were designed for each experiment. Duncan’s multiple range tests were performed, and the significant differences at a significance level of *p* < 0.05 were indicated by different letters assigned to each value.

The relative expression levels of *TT16-3* are much higher than those of *AGL8* in the early, middle, and late germination stages (Figure 7D,E). Notably, their expression levels are significantly increased in both the middle and late germination stages (Figure 7D,E), suggesting their potential involvement in germination processes following embryo metabolic activity. To further validate the expression level of *AGL8* and *TT16-3* in male flowers, qRT-PCR experiments were fulfilled for five stages of flower development, ranging from small flower buds to mature flowers (Figure 8A,B).

The expression levels of *AGL8* were higher than those of *TT16-3* during male flower development (Figure 8C,D).

## 3. Discussion

In this study, we characterized the MADS-box Type II gene family in papaya and gained insights into their roles in plant growth, development, and stress responses. We identified 24 Type II genes in papaya, which were grouped into 12 subclades based on phylogenetic analysis. The smaller number of genes in papaya compared to *A. thaliana* and *V. vinifera* is likely due to gene loss events throughout papaya evolution, which is consistent with the compact gene content in papaya [53]. The intron–exon structure and conserved motif organization were mainly conserved within the same subclade, indicating their common evolutionary origin.

The expression patterns of the Type II genes in specific tissues reflect their diverse roles in organ development. For instance, the elevated expression of *AGL18* in male flowers and pollen suggests that it may promote male floral organ differentiation. Several genes were found to be upregulated under abiotic and biotic stresses, suggesting their involvement in stress responses. Among them, *AGL19* consistently showed a high expression under drought and chilling stresses, highlighting its potential role in stress acclimation. Notably, several Type II genes showed differential expression between male and female flowers, indicating their association with sex determination and flower development in papaya. These genes’ upstream cis-elements and co-expression modules suggest that they are involved in hormone metabolism and stress responses. The expression patterns of *AGL8* and *TT16-3* in male flowers and seed germination were validated via qRT-PCR. Overall, these findings provide evidence for the diverse functional roles of Type II genes in papaya, particularly in organ development, stress responses, and sex determination.

Duplicated genes arise through evolutionary mechanisms such as genome-wide duplication, homologous recombination, or transposon events, resulting in genes with extra copies in the genome [39,40]. Gene duplication was the primary mechanism for the formation of the MADS-box Type II subfamily in papaya [41]. These duplicated genes may have similar functions, leading to the masking of phenotypic consequences of mutations in one gene by another, a phenomenon referred to as gene functional redundancy [41]. The observed expression divergence between most duplicated gene pairs implicates functional diversification after duplication [54]. Previous research have shown that gene duplication is common among other transcription factors and species [44]. For example, in tomato, five pairs of WRKY genes were found to have originated from tandem duplicate events [55], and in the Cavendish banana genome, nine tandem duplicated gene pairs were identified in the bHLH transcription factor family [56]. In our study, only one tandem duplicated pair *AP3-1* and *AP3-2* was observed in papaya, which is consistent with its compact gene set [3,53]. These findings suggest that the recent gene duplications may be prevalent in flowering plants, but are inactive in papaya. The conserved expression bias of *AGL19* and *AGL2* in different transcriptomes provided a case study of subfunctionalization between duplicated genes. In contrast, the changing expression dominance of *AP1* and *AGL8* in different conditions implied neofunctionalization. These findings demonstrate the complex expression evolution of duplicated Type II genes in papaya.

In conclusion, the study provides novel insights into the evolution and expression diversity of the MADS-box Type II gene family in papaya, and identifies candidate genes that may play pivotal roles in plant growth, stress responses, and the development of different sex-type flowers in papaya [11]. Further functional characterization of these genes will contribute to a deeper understanding of the molecular mechanisms underlying papaya development and the regulation of its sexuality.

## 4. Materials and Methods

### 4.1. Identification of MADS-Box Type II Genes

The MADS-box family HMM model file SRF-TF (PF00319) was downloaded from the Pfam database [57]. The candidate MADS-box Type II genes were aligned to HMM file by hmmsearch with default parameters to filter out the candidates without the MADS-box domain [58]. The retained candidate genes were analyzed in NCBI-CDD online databases to verify the existence of MADS domain [59]. Amino acid sequences that contain the SRF-TF domain were obtained and then used BLASTP (V 2.5.0) against the Protein database among *Arabidopsis thaliana*, *C. papaya*, and *Vitis vinifera*. Furthermore, iTAK v1.6 was used to identify the genome-wide type II MADS-box genes for *Arabidopsis thaliana*, *Carica papaya*, and *Vitis vinifera*, respectively [17].

### 4.2. Phylogenetic Tree Construction

A total of 90 MADS-box Type II protein sequences obtained from *C. papaya*, *A. thaliana*, and *V. vinifera* were used for phylogenetic tree building. Multiple sequences alignment was performed using muscle v3.8.31 with default parameters [60]. A maximum-likelihood (ML) phylogenetic tree of these MADS-box Type II genes was constructed using IQ-TREE (V.2.1.2) with the parameters ‘-redo -bb 1000 -mset raxml -m TEST’ [28]. Finally, the phylogenetic tree was visualized using iTOL v6 (https://itol.embl.de/, accessed on 15 August 2023).

### 4.3. Chromosomal Location, Exon/Intron Structure, and Chromosomal Location Analyses

The positions of MADS-box Type II genes were obtained from the genome annotation file (gff). The online program MG2C v2.1 was applied to draw their physical locations on nine chromosomes [61]. The exon/intron structures of MADS-Type II genes were displayed using the gene structure display server (GSDS 2.0) program [23]. The protein sequences were submitted to MEME online tool for motif prediction [37]. The predicted motifs were visualized using TBtools [62].

### 4.4. Physicochemical Properties of MADS-Box Type II Genes

Expasy [18] was used to predict protein isoelectric point and molecular weight of the MADS-box Type II genes. The physicochemical properties of MADS-box Type II genes in *C. papaya* were obtained. The subcellular localization of the MADS-box Type II genes was predicted via WoLF PSORT [19] and Cell-PLoc 2.0 [63].

### 4.5. Duplicated Genes Analysis

The duplicated genes of type II MADS-box genes within species were identified using DupGen_finder [44]. Fifteen duplicated pairs of MADS-box Type II genes were identified in the papaya genome, including ten transposon duplicated genes, one proximal duplicated gene, two whole-genome duplicated genes, and two dispersed duplicated genes.

To analyze selection pressure and estimate the time of duplication events, the protein sequences of each duplicated gene pair were aligned using Muscle v3.8.31 [60] and retranslated into coding sequences (cds) using ParaAT v2.0 [64]. Then, the non-synonymous substitution rate (Ka), synonymous substitution rate (Ks), and the ratio of non-synonymous to synonymous values (Ka/Ks) of the duplicated gene pairs were calculated using KaKs_Calculator v2.0 [65]. The occurrence time of each duplicated gene pair was calculated, using the formula: T= Ks/2μ. Of which, μ means the mutation rate of *C. papaya* per site per year (μ = 7.22 × 10${}^{-9}$).

To investigate synteny, the internal homologous gene blocks of the papaya, and the collinear relationships among *A. thaliana*, *C. papaya*, and *V. vinifera* were investigated using Mcscan software [66]. The links between duplicated genes and genomic internal syntenic blocks were visualized using Circos (v0.69-8) [67].

### 4.6. Gene Expression Analysis

The transcriptome data of different tissues (flower, stamen, pistil, pollen, and leaf), under abiotic stress (drought stress, chilling stress, and hormone stress) and pathogen infection (*Papaya Mosaic virus*, *Papaya Ringspot virus*, and *Colletotrichum brevisporum*) were downloaded from the NCBI SRA repository. A total of 152 Gb RNAseq data from 152 samples were used for transcriptomic analysis (Table S6). The fastp program [68] was used to perform adapter trimming and quality control with default parameters. The clean reads were aligned to the reference genome using HISAT2 v1.34d [69], and then transcripts were assembled and gene expression levels were calculated using StringTie v2.1.0 [70]. DESeq2 was used to identify differentially expressed genes with cutoff values of FDR ≤ 0.05 and |fold change| ≥ 2 [71].

### 4.7. Profiling the Expression Patterns between Male and Female Flowers

The Mfuzz R package’s fuzzy c-means method was used to profile MADS-box Type II genes based on their expression patterns [72]. Mfuzz analysis was performed on the genes that differed in expression between male and female groups at each time point. The input was the average FPKM value for each gene at each developmental time point (flower and pistil). Following standardization, each gene was allocated to a distinct cluster based on its membership value. The clusterProfiler package was used to perform functional enrichment analysis on each cluster [73].

### 4.8. Cis-Regulatory Element Analysis

To predict the cis-elements in the upstream (2 kb) of duplicated *AGL8* and *TT16-3*, we submitted these sequences to the PlantCARE database [74]. The cis-element distribution was visualized using Tbtools [62]. We utilized the FIMO online software [37] (https://meme-suite.org/meme/tools/fimo, accessed on 20 August 2023) to identify transcription factor binding sites (TFBSs) in the promoters of papaya MADS-box Type II genes, with a motif recognition threshold set at a *p*-value less than 0.0001. By comparing them with TFBS motifs generated from all papaya protein-coding genes, we conducted enrichment analysis of TFBS motifs for 24 MADS-box type II genes. Fisher’s exact test was employed to estimate *p*-values. TFBS motifs with adjusted *p*-values below 0.05 were considered as putative regulatory transcription factors (TFs) for papaya type II genes (Table S11). The plant transcription factor motif file was obtained from the JASPAR database [75] (https://jaspar.genereg.net/download/data-/2022/CORE/JASPAR2022_CORE_non-redundant_pfms_meme.txt, accessed on 25 August 2023). We performed filtering based on the “Taxon” information provided in the detailed description of each motif. As a result, 1298 motifs that did not belong to plants were filtered out, leaving us with 658 plant-specific motifs to be used as input files for FIMO prediction.

### 4.9. Functional Enrichment Analysis

Gene Ontology (GO) and Kyoto Encyclopedia of Genes and Genomes (KEGG) pathway enrichment analyses were performed using the clusterProfiler R package [73]. Pathways and terms having a Benjamini–Hochberg-adjusted *p*-value less than 0.05 was considered significantly enriched.

### 4.10. Weighted Gene Co-Expression Network Analysis

Weighted gene co-expression network analysis (WGCNA) was performed using differentially expressed genes (DEGs) between female and male flowers during three development stages. The co-expression network for these DEGs was constructed using the WGCNA R package (v1.69) [76], with “cv = 0.3, fold = 0.3, and other default parameters”. Modules significantly associated with male or female flower development stages were selected for further analysis. Go term and KEGG pathway enrichment analyses were performed using clusterProfiler v4.0 [73] for genes within the interested modules. The hub-networks of hub-genes intensely co-expressed in cyan module was constructed and visualized using Cytoscape v3.9.1 [77].

### 4.11. qRT-PCR Analysis of AGL8 and TT16-3

Samples were obtained from whole seeds at three distinct germination phases, namely early, medium, and late germination processes, for quantitative real-time polymerase chain reaction (qRT-PCR) analysis. The seed becomes larger, absorbs water, the exterior seed shell softens, and the seed embryo starts to metabolically function at the early stage known as activation (Figure 7A). The embryo breaks through the seed shell, the torpedo-shaped embryo becomes visible, and the embryonic root develops during the middle stage, which is characterized by radicle emergence (Figure 7B). The seed embryo starts to germinate and the embryonic shoot fully emerges at the late stage of shoot emergence (Figure 7C). Additionally, samples from five development stages of papaya male flowers, ranging from small flower bud to mature flower, were collected for qRT-PCR analysis (Figure 8A,B). RNAprep pure polysaccharide and polyphenol plant total RNA extraction kit (DP441, TIANGEN, Beijing, China) was used to extract the RNA of papaya seeds in the early, middle, and late stages of germination. The extracted RNA was then reverse-transcribed using Tolo biotech kit ToloScript RT SelectMix for qPCR (+2-Step gDNA Erase-Out, TOLOBIO, Shanghai, China).

Using the program Primer 5, primers for qRT-PCR genes were built. The product length was regulated between 80 and 300 bp, and the Tm value range was kept between 58 and 60 °C. The specific primers were selected for synthesis (Table S14). Real-time fluorescence quantitative PCR reaction was performed using Novozan quantitative kit, Real-time fluorescence quantitative PCR reactions were performed using the Novozan quantitative kit called ChamQ Universal SYBR qPCR Master Mix (Vazyme, Nanjing, China), and the qTOWER³auto (230 V) 96-well automatic qPCR unit was used for PCR instrument. The quantitative results were analyzed using the calculation formula: $2^{-▵▵\mathrm{Ct}}$ with the internal reference gene of papain in papaya. The PCR cycling parameters were as follows: 95 °C for 30 s, 40 cycles of 95 °C for 10 s, 60 °C for 30 s, 95 °C for 15 s, 60 °C for 60 s, and 95 °C for 15 s. Three biological replicates were included in each qRT-PCR experiment conducted for both seed germination and male flower development processes.

## Figures and Tables

**Table 1:** Physicochemical properties’ analysis of 24 MADS-box Type II proteins in *C. papaya*.

Gene ID	Name	Isoelectric Point	Molecular Mass/Da	Amino Acid/aa	* (Subcellular Localization)			
					Nucl	Mito	Chlo	Plas
Cpa02g009980.Q8	*TT16-1*	5.48	27,605.07	236	3			2
Cpa02g010140.Q8	*TT16-2*	6.21	29,715.62	254	4	6	3	1
Cpa06g013660.Q8	*TT16-3*	6.46	27,808.64	233	6	4	2	1
Cpa01g023480.Q8	*PI*	9.2	24,739.33	210	14			
Cpa04g004320.Q8	*AP3-2*	9.43	26,288.85	227	14			
Cpa04g004350.Q8	*AP3-1*	9.36	26,493.15	227	14			
Cpa09g006730.Q8	*AGL20*	9.88	24,036.64	207	8	1		1
Cpa04g006820.Q8	*AGL19*	9.26	21,586.97	186	12			
Cpa06g007810.Q8	*AGL21-1*	9.53	27,734.85	239	14			
Cpa01g014370.Q8	*AGL21-2*	9.52	29,027.34	249	6	3.5	3	1
Cpa08g009630.Q8	*AGL18*	8.91	27,028.72	235	14			
Cpa08g001780.Q8	*AGL15*	8.97	27,722.44	246	11		1	1
Cpa04g001320.Q8	*SVP*	8.32	25,858.39	227	14			
Cpa01g017880.Q8	*AGL24*	8.73	26,646.57	237	14			
Cpa01g017490.Q8	*AGL12*	5.88	22,542.21	199	3	5	1	1.5
Cpa01g014320.Q8	*STK*	9.33	25,229.84	219	14			
Cpa03g022480.Q8	*SHP1*	9.24	30,265.32	260	14			
Cpa09g003660.Q8	*AGL8*	8.97	27,497.17	238	14			
Cpa02g016330.Q8	*AP1*	8.77	28,238.26	244	14			
Cpa05g006580.Q8	*AGL6*	9.2	28,078.07	247	14			
Cpa06g017170.Q8	*SEP3-1*	8.79	27,757.67	243	14			
Cpa01g006740.Q8	*AGL2*	9.08	28,325.32	248	14			
Cpa09g003210.Q8	*AGL4*	8.59	27,410.31	239	14			
Cpa02g016320.Q8	*SEP4*	8.34	28,103.09	243	14			

* Subcellular localization: refers to the specific location of a protein within a cell. Nucl: The nucleus is a prominent organelle in eukaryotic cells that contains the genetic material of the cell. Mito: Mitochondria are organelles that are responsible for energy production in eukaryotic cells. Chlo: Chloroplasts are organelles found in plant and algal cells that are responsible for photosynthesis. Plas: The plasma membrane is the outermost layer of the cell that separates the interior of the cell from the extracellular environment.

**Table 2:** Selective pressure (Ka/Ks) of MADS-box Type II genes derived from gene duplication process.

Duplicated Gene Pairs	Duplicated Types	Sequence Similarity (%)	Ka	Ks	(Ka/Ks)	Time of Duplication Events (Mya)
*AP3-1/AP3-2*	proximal	92.07	0.047	0.204	0.231	1.4
*TT16-3/AGL8*	transposed	41.624	0.685	3.672	0.187	25.4
*SEP3-1/AGL2*	transposed	66.932	0.229	2.719	0.084	18.8
*TT16-2/AGL8*	transposed	33.891	0.681	3.710	0.183	25.7
*TT16-1/AP1*	transposed	33.696	0.681	3.707	0.184	25.7
*AGL12/SHP1*	transposed	46.893	0.520	3.626	0.143	25.1
*AGL19/AGL2*	transposed	47.682	0.458	3.496	0.131	24.2
*AGL20/SHP1*	transposed	36.585	0.642	3.679	0.174	25.5
*AGL4/AGL2*	transposed	62.4	0.214	1.746	0.123	12.1
*AGL18/AGL21-1*	transposed	47.468	0.557	3.736	0.149	25.9
*AGL15/AGL6*	transposed	51.572	0.557	3.775	0.147	26.1
*AP1/AGL8*	wgd	70.423	0.262	1.506	0.174	10.4
*AGL24/SVP*	wgd	62.979	0.231	1.641	0.141	11.4
*TT16-1/TT16-2*	dispersed	92.520	0.359	3.731	0.096	25.8
*TT16-2/TT16-3*	dispersed	45.106	0.002	* NA	NA	NA

* NA: No corresponding result was obtained.

## Data Availability

Not applicable.

## Supplementary Materials

The following supporting information can be downloaded at: https://www.mdpi.com/article/10.3390/ijms241814039/s1.
