# Supplementary material for: Genomic and Transcriptomic Insights into the Evolution and Divergence of MIKC-Type MADS-Box Genes in Carica papaya
Source: Int J Mol Sci. 2023 Sep 13;24(18):14039. doi: 10.3390/ijms241814039 (PMC10531014; doi:10.3390/ijms241814039)
Supplement: Supplementary file 1 [file ijms-24-14039-s001.zip › Supplemental_Figures.pdf]

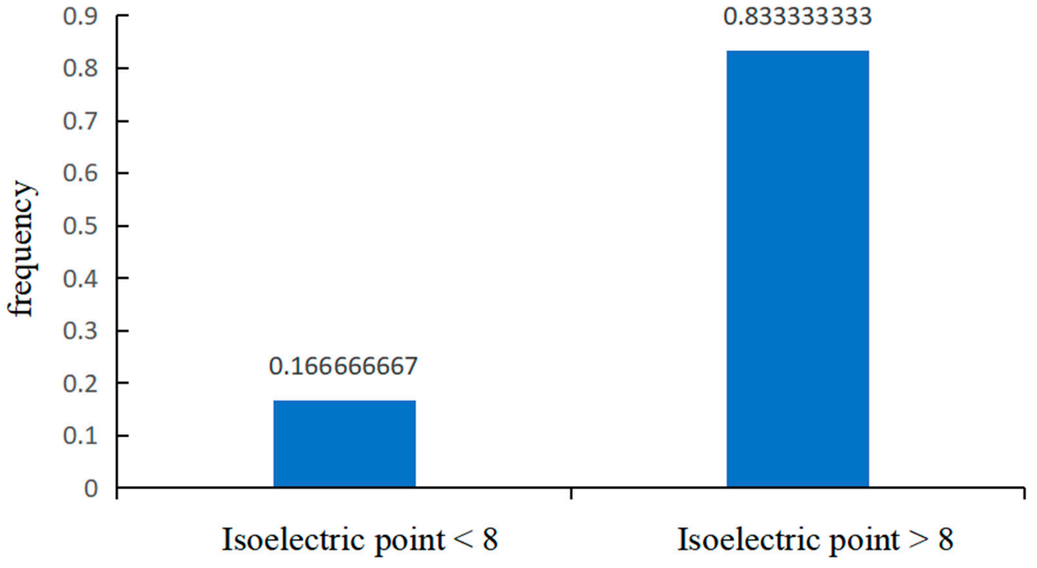

Figure S1. Histogram of frequency distribution of isoelectric points of 24 MADS-box Type II genes.

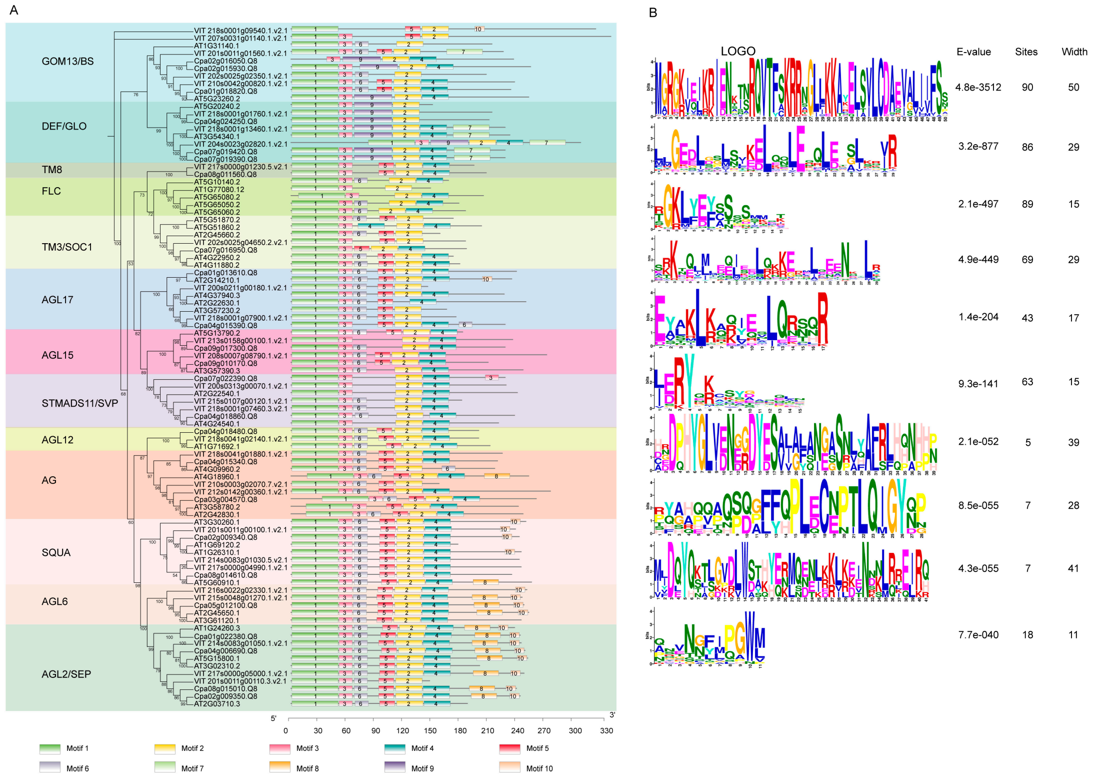

Figure S2. Conserved motifs of MADS-box Type II proteins were analyzed using MEME suit. (**A**) Theorganization of conserved motifs in MADS-box Type II gene. (**B**) A SeqLogo made up of ten conserved motifs for each subfamily. Sequence logo with an E-value smaller than 7.7e-040 was chosen.

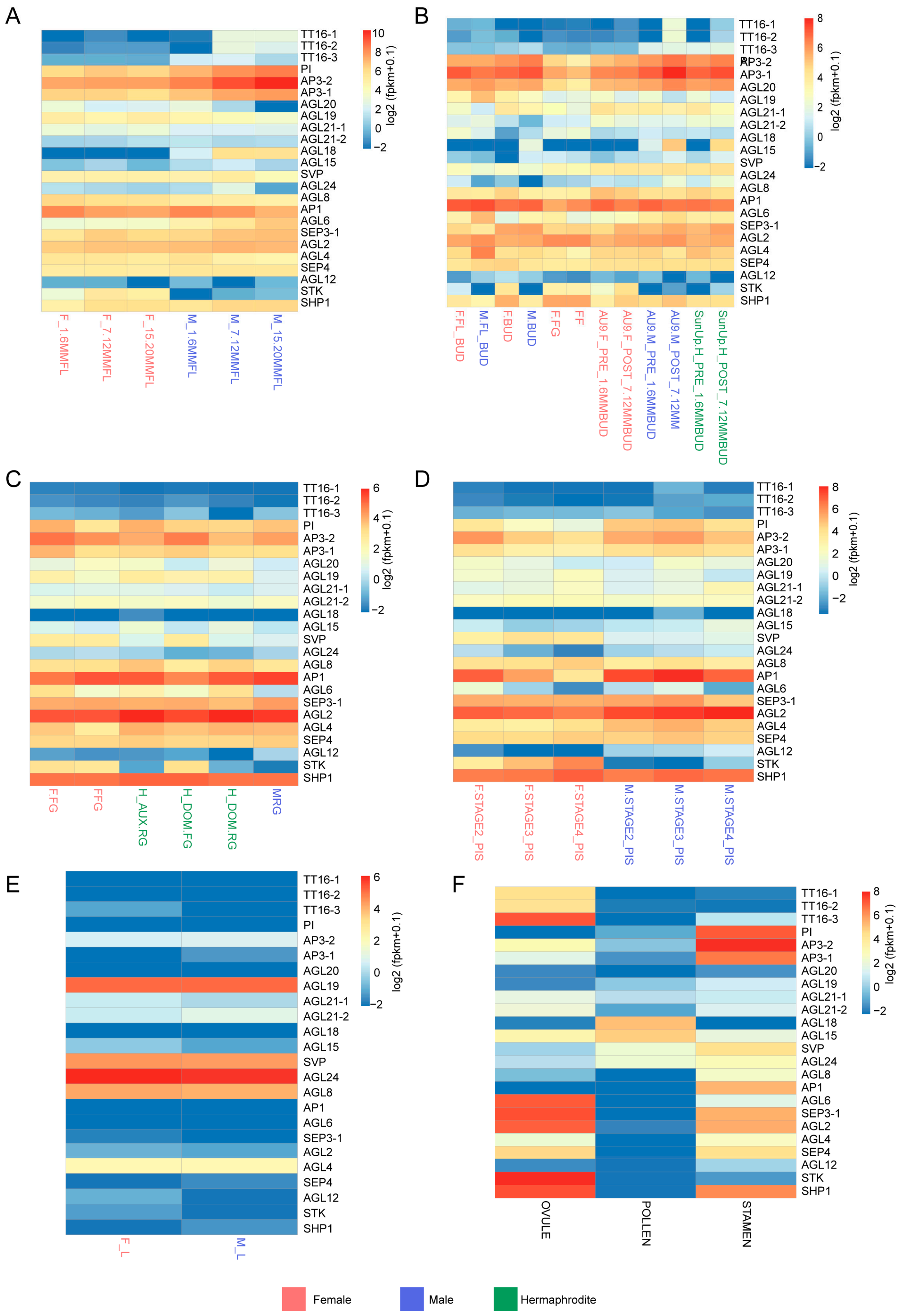

Figure S3. Expression profiles of 24 MADS-box Type II genes in flowers (**A**), flower buds (**B**), gynoecia (**C**), pistils (**D**), leaves (**E**), ovules, pollens and stamens (**F**). The detailed sample information were provided in Table S6.

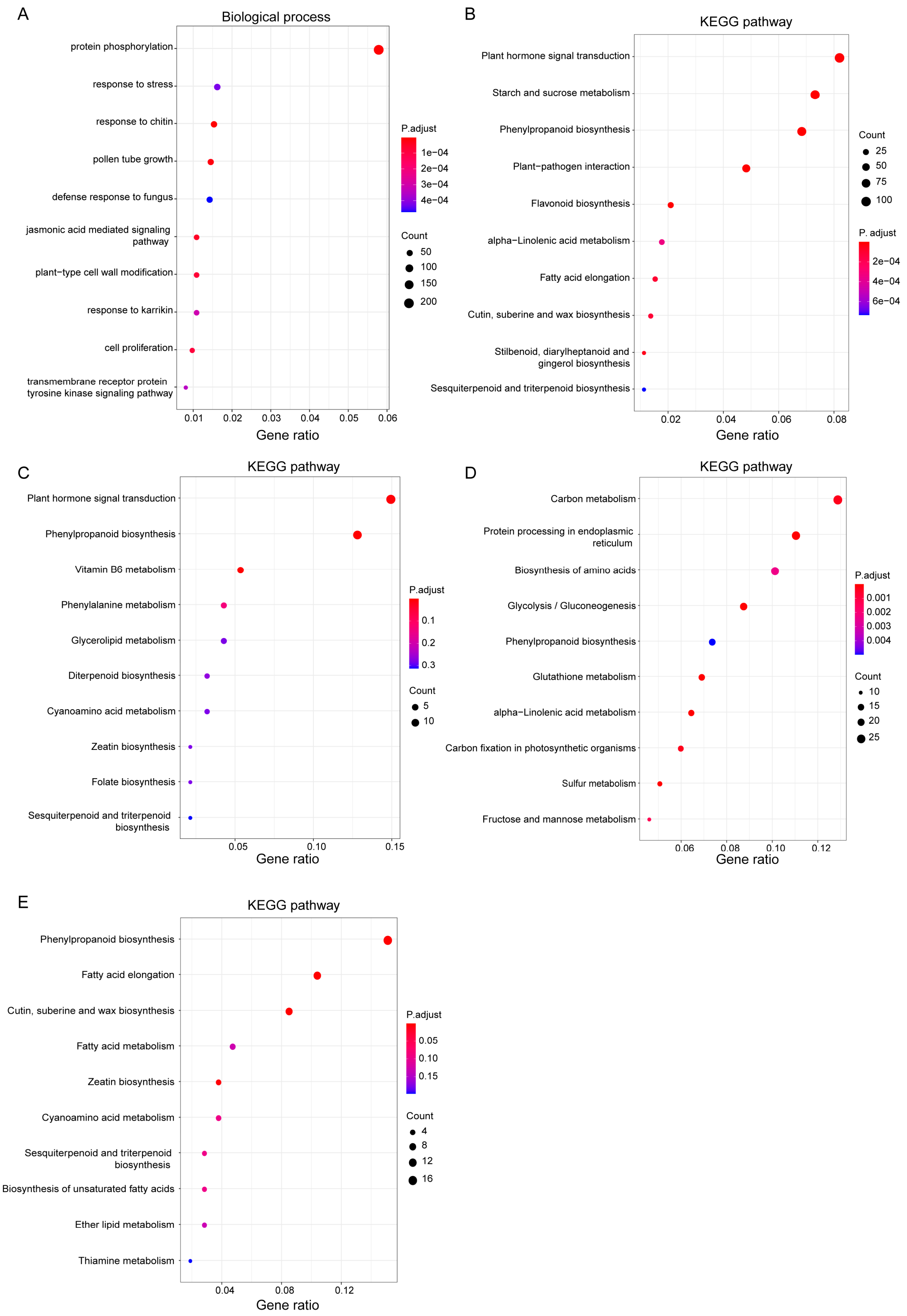

Figure S4. Gene ontology (GO) annotation (**A**), and Kyoto encyclopedia of genes and genomes (KEGG) pathway enrichment analysis (**B**) of the genes in 7,159 DEGs between papaya male and female flowers. (**C**) Kyoto encyclopedia of genes and genomes (KEGG) pathway enrichment analysis of the genes in cyan module generated by WGCNA analysis. (**D**) KEGG pathway enrichment analysis of genes having same expression trend in Cluster-

7. (**E**) KEGG pathway enrichment analysis of genes having same expression trend in Cluster-9.

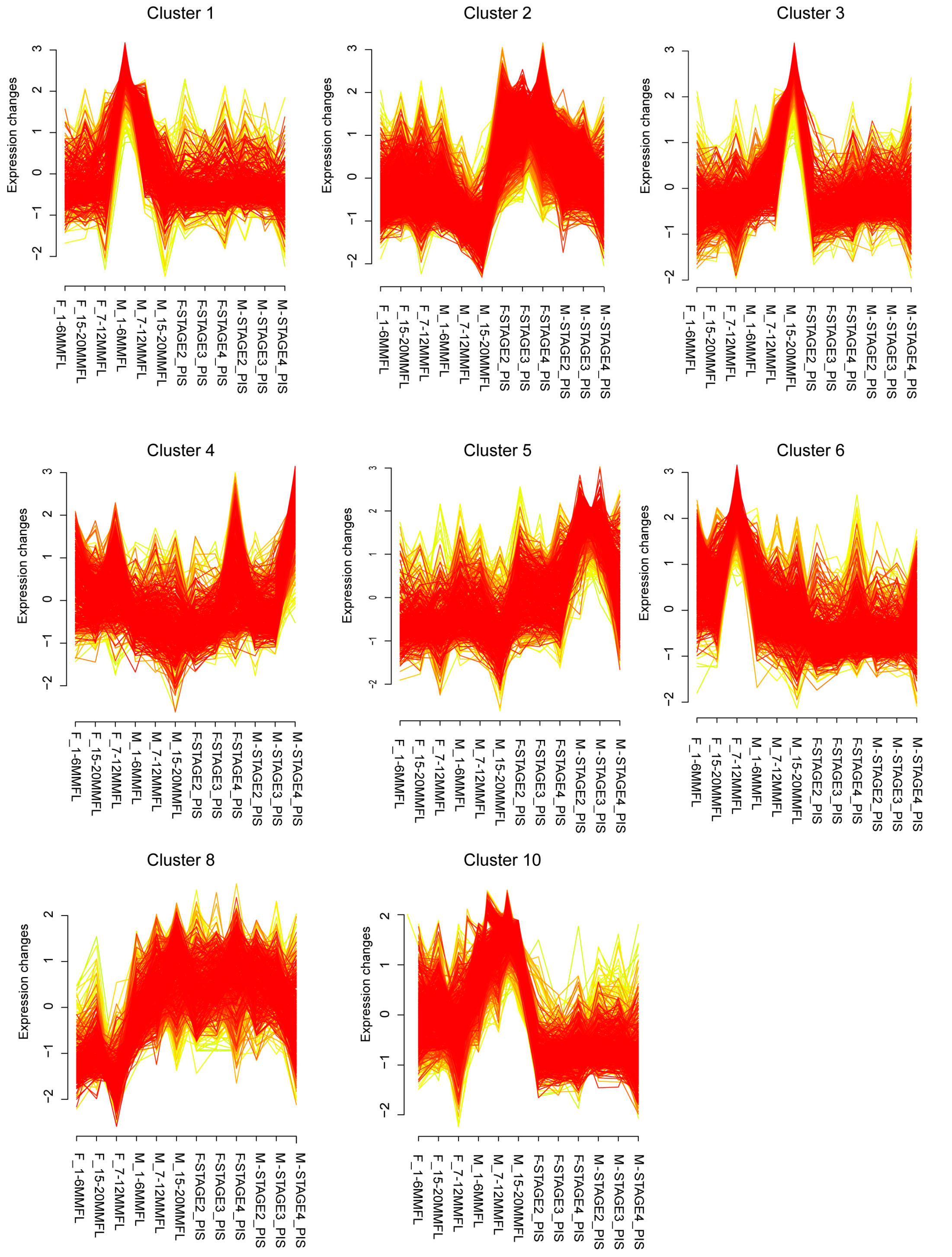

Figure S5. The expression patterns of 7,159 DEGs in three development stages of male- and female- flowers. The clustering analyze of time-resolved transcriptomes for different flower development stages was performed using Mfuzz package. The Cluster-7, and Cluster-9 were placed in Figure 5.

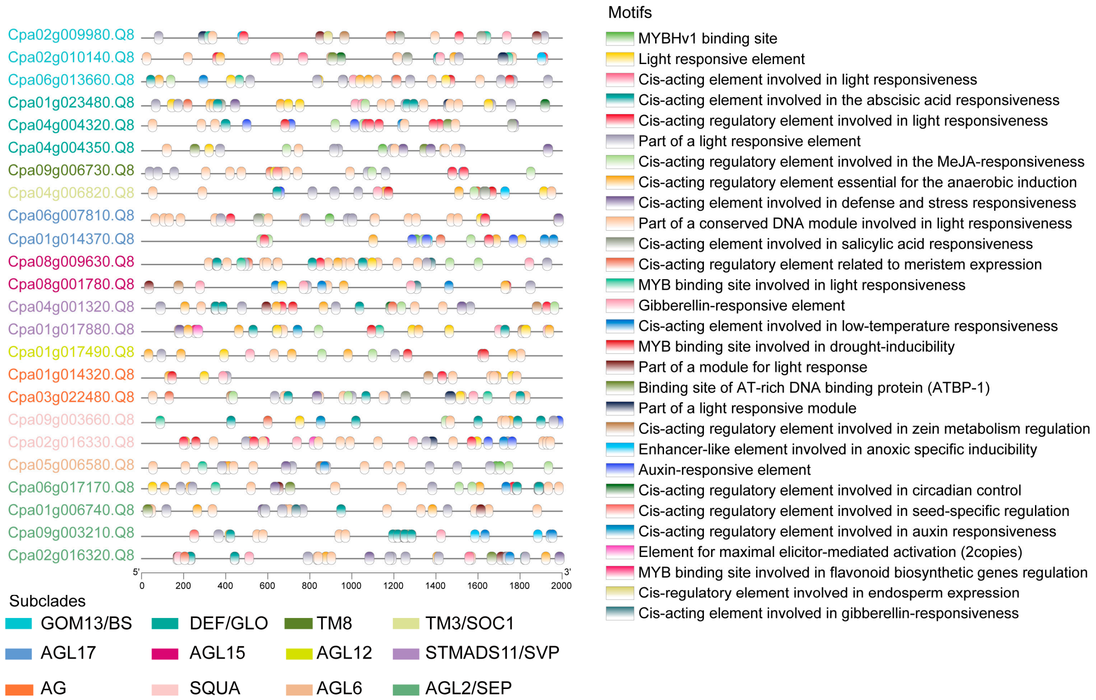

Figure S6. Analysis of cis-regulatory elements for 24 MADS-box Type II genes in papaya.

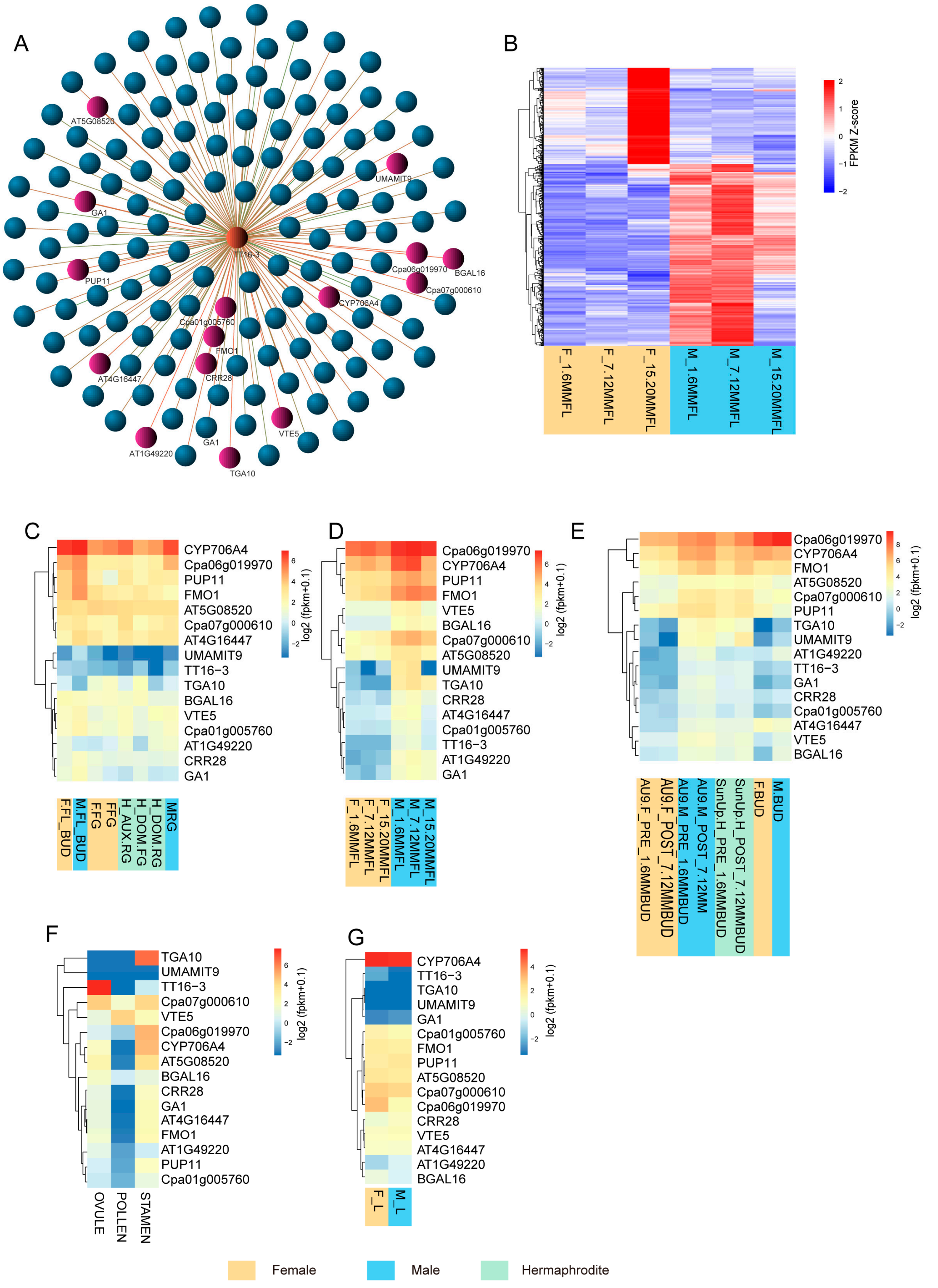

Figure S7. Expression of genes co-expressed with *TT16-3* in three developmental stages of male and female flowers. (**A**) Co-expression network of genes directly associated with *TT16-3* in the cyan module. (**B**) Expression profile of genes in cyan module which involved in male- or female- flower development. Expression heatmap of 15 genes highly associated with *TT16-3* were analyzed by WGCNA in different tissues of flower buds (**C**), flowers (**D**), gynoecia (**E**), ovules, pollens, stamens (**F**) and leaves (**G**).
